# Non-echoplanar diffusion weighted imaging in the detection of post-operative middle ear cholesteatoma: navigating beyond the pitfalls to find the pearl

**DOI:** 10.1007/s13244-016-0516-3

**Published:** 2016-08-24

**Authors:** Ravi K. Lingam, Robert Nash, Anooj Majithia, Ali Kalan, Arvind Singh

**Affiliations:** 1 Department of Radiology, Northwick Park and Central Middlesex Hospitals, London Northwest Healthcare NHS Trust, London, England UK; 2 Department of Otolaryngology, Head & Neck Surgery, Northwick Park and Central Middlesex Hospitals, London Northwest Healthcare NHS Trust, London, England UK

**Keywords:** Diffusion weighted imaging, MRI, Cholesteatoma, Pitfalls, DWI

## Abstract

**Abstract:**

Non-echoplanar diffusion weighted magnetic resonance imaging (DWI) has established itself as the modality of choice in detecting and localising post-operative middle ear cleft cholesteatoma. Despite its good diagnostic performance, there are recognised pitfalls in its radiological interpretation which both the radiologist and otologist should be aware of. Our article highlights the various pitfalls and provides guidance for improving radiological interpretation and navigating beyond many of the pitfalls. It is recommended radiological practice to interpret the diffusion weighted images together with the ADC map and supplement with the corresponding T1 weighted and T2 weighted images, all of which can contribute to and enhance lesion localisation and characterisation. ADC values are also helpful in improving specificity and confidence levels. Given the limitation in sensitivity in detecting small cholesteatoma less than 3 mm, serial monitoring with DWI over time is recommended to allow any small residual cholesteatoma pearls to grow and become large enough to be detected on DWI. Optimising image acquisition and discussing at a joint clinico-radiological meeting both foster good radiological interpretation to navigate beyond the pitfalls and ultimately good patient care.

***Teaching Points*:**

• *Non-echoplanar DWI is the imaging of choice in detecting post-operative cholesteatoma.*

• *There are recognised pitfalls which may hinder accurate radiological interpretation.*

• *Interpret with the ADC map /values and T1W and T2W images.*

• *Serial DWI monitoring is of value in detection and characterisation.*

• *Optimising image acquisition and discussing at clinico-radiological meetings enhance radiological interpretation.*

## Introduction

Since it was first described in 2006 for the detection of cholesteatoma [1, 2], non-echoplanar diffusion weighted magnetic resonance imaging (DWI) has now firmly established its role as the imaging modality of choice in detecting post-operative cholesteatoma [3–7]. It has superseded CT, echoplanar DWI and delayed contrast MRI by virtue of its superior diagnostic performance [3–8]. Non-echoplanar DWI is capable of acquiring thin slices (as thin as 2 mm) and generating a high resolution matrix, and hence can detect cholesteatoma as small as 2 mm [3–5]. It also has the advantage of not requiring intravenous gadolinium contrast, which is implicated with nephrogenic systemic fibrosis [9] and intracerebral deposition [10]. It also does not require delayed scans as with delayed contrast MRI [3]. Even though echoplanar DWI has a shorter acquisition time, non-echoplanar DWI performs better than its echoplanar counterpart primarily due to the lack of air-bone susceptibility artefact and distortion at the temporal bones [3, 6]. It offers the potential to reduce the number of mandatory second-look (or re-look) surgery in detecting residual or recurrent disease [3, 5, 7]. However, the limitations of the technique still need to be considered as part of a safer and cost-effective work-up strategy. This article aims to highlight pitfalls of radiological interpretation and empower the reader with necessary approaches to navigate beyond the pitfalls.

## Optimisation of technique

**Fig. 1:**
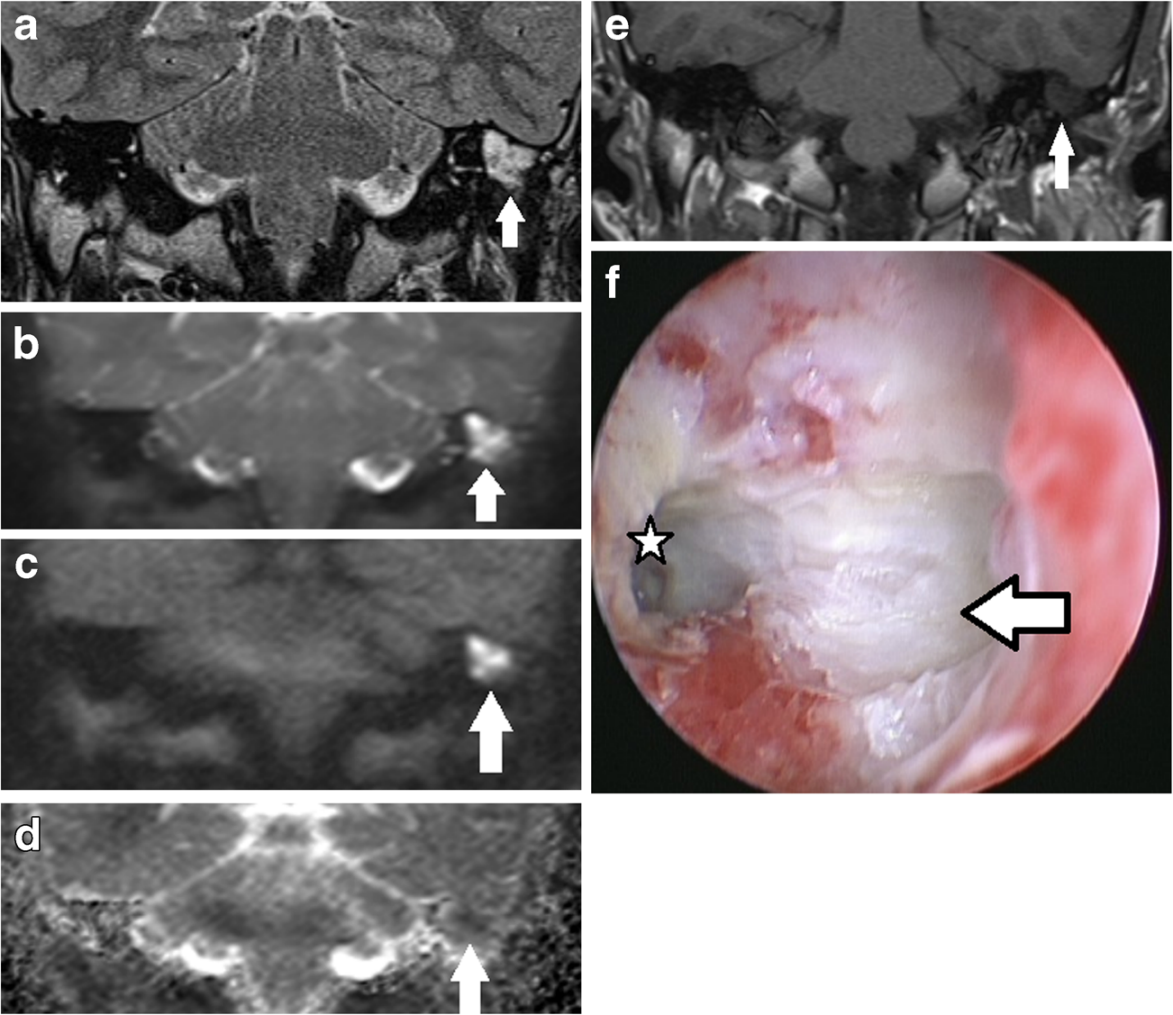
Typical MRI characteristics of post-operative cholesteatoma. Coronal images through the left mastoid remnant shows the lesion (*white arrow*) as high signal on **a** the T2 weighted image, **b** DWI b0 image, and **c** DWI b1000 image and a low signal on the **d** ADC map and **e** non-contrast enhanced T1 weighted image. **f** Surgery confirms the presence of the cholesteatoma. The keratin has been suctioned away showing the underlying white squamous epithelium lining the mastoid remnant (*white arrow*) and aditus (*star*)

**Fig. 2:**
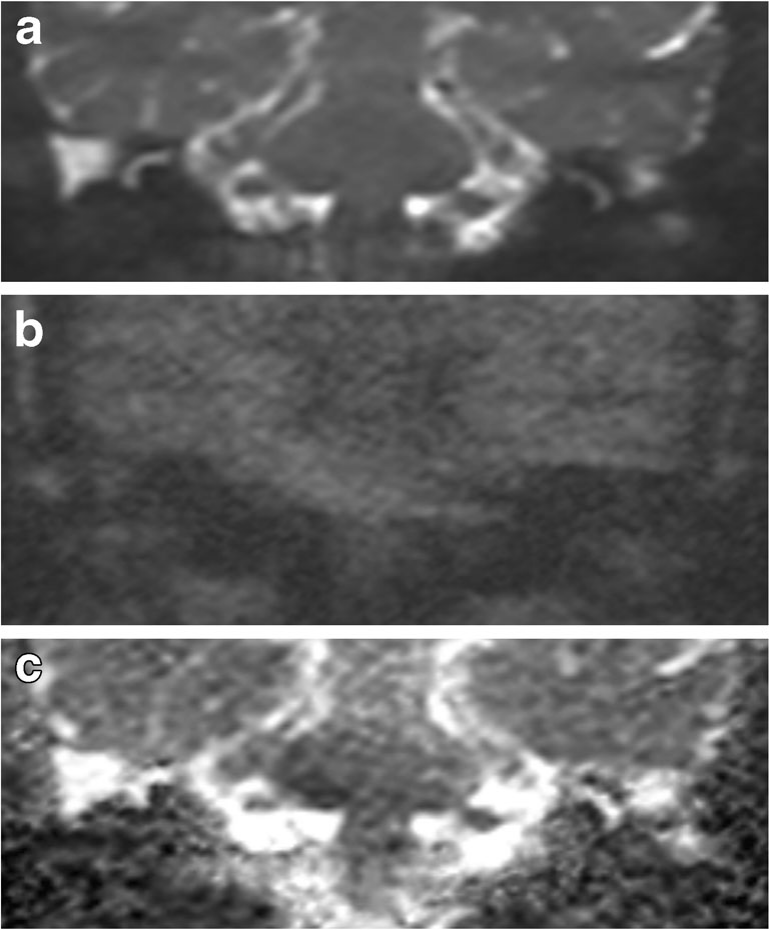
Typical DWI appearance of non-cholesteatomatous soft tissue. Coronal images through the right post-operative mastoid remnant shows the soft tissue (*white arrow*) as high signal on **a** the b0 image, but loses signal on **b** the b1000 image, and hence demonstrates high signal on **c** the ADC map. Surgery confirms granulation tissue

By virtue of its keratin content, cholesteatoma returns high signal intensity compared to brain tissue on the diffusion weighted images obtained both at b values of 0 s/mm^2^ and at higher values of 800 or 1000 s/mm^2^ (Fig. 1). This is due to a combination of restricted diffusion and T2 shine-through effect. It returns low signal on the apparent diffusion coefficient (ADC) map [3, 4, 9]. The ADC map is free from any T1 or T2 effects, provides a true quantitative display of water diffusivity at each voxel [11], and is integral in the interpretation of the DWI images [3, 12]. Non-cholesteatomatous soft tissue such as granulation tissue, inflammation, and fluid return lower or no signal on the high b value (b800 or b1000) images compared to the b0 images and consequently high signal on the ADC map (Fig. 2).

**Fig. 3:**
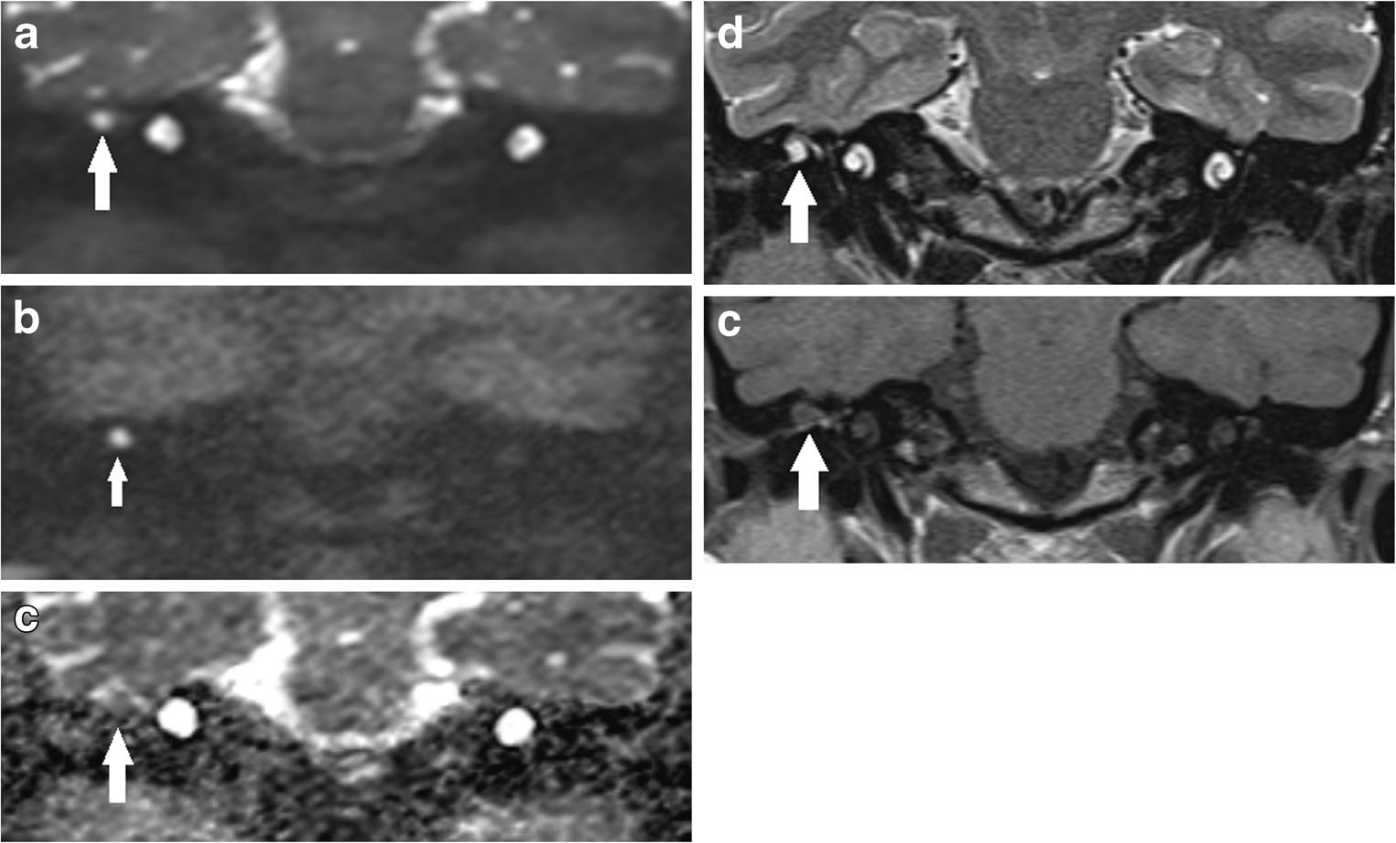
DWI appearance of a small residual cholesteatoma confirmed on second-look surgery. The **a** b0 and **b** b1000 image shows a small 5 mm high signal cholesteatoma (*white arrow*) at the right epitympanum. It returns low signal on **c** the corresponding ADC map, high signal on **d** the T2 weighted image and low signal on **e** the non-contrast enhanced T1 weighted image

To allow accurate radiological interpretation, the DWI images need to be optimal in the first instance. The non-echoplanar technique can generate image slices without air-bone susceptibility artefacts and distortion, as thin as 2–3 mm producing a high resolution matrix, which is essential for the detection of tiny cholesteatoma pearls (Fig. 3). Current non-echoplanar sequences broadly include single-shot turbo spin-echo (SSTSE) sequences [such as HASTE (half Fourier SSTSE) [13–19]] and multishot turbo spin-echo (MSTSE) sequences [such as with PROPELLER (periodically rotated overlapping parallel lines with enhanced reconstruction) [20–23]]. Currently, slice thickness as low as 2 mm can be acquired with HASTE and 3 mm with PROPELLER DWI [4]. The SSTSE sequence such as HASTE acquire one b value at a time (currently 5 to 6 min for each b value) and are susceptible to both movement artefacts and slice position misregistration errors in post-acquisition ADC map calculation. In contrast, the multidirectionally acquired sequences, however, can acquire two b values in a single acquisition (currently under 5 min with multishot PROPELLER DWI), and hence theoretically quicker and less susceptible to motion artefacts and slice position misregistration errors than HASTE [4]. The MSTSE sequences have a shorter echo train than their single shot counterparts and hence are able to mitigate the T2 blurring effects (image degradation due to T2 decay during acquisition) [24]. As a SSTSE sequence, but using a half-Fourier acquisition, HASTE is also able to reduce the effect of T2 blurring. With most types, non-echoplanar diffusion weighted images can be acquired in any plane. Currently, with the PROPELLER DWI sequence, the images can currently only be acquired in the axial plane [3, 4, 20, 23], but it can be reconstructed in other planes [21]. Three dimensional (3D) DWI acquisition with multiplanar reconstruction has also been described with TFE-DSDE [Turbo field-echo with diffusion-sensitized driven equilibrium] [25]. In our institution, the coronal images are preferred as it demonstrates the tegmen and the relevant tympanomastoid anatomy better for more confident localisation of disease and surgical planning. Ideally, the DWI sequences should be supplemented by conventional T2 weighted and T1 weighted images of similar slice thickness and slice positions to the DWI images to obtain the anatomical information required for better localisation of disease. On conventional MRI, cholesteatoma typically returns high signal on the T2 weighted MR images and low signal on the T1 weighted MR images (Fig. 1) [3]. Though not specific, these sequences, together with the DWI sequences, can help further characterise the disease and detect cholesteatoma more accurately [4]. Supplementing with post-gadolinium enhanced contrast scan is not necessary as it does not confer greater diagnostic performance than non-echoplanar DWI alone [8].

Typically, two b values are sufficient to generate the ADC map, which is reconstructed following acquisition of the scans [12]. More b values can be used to strengthen further the ADC map, but would incur a longer scanning time and a higher risk of patient movement during scanning and image misregistration. Crucially, for non-multidirectionally acquired sequences (such as HASTE), which require separate acquisitions for each b value, the measurement parameters and the slice positions for the various b values have to be identical (copy-referenced) to promote better image registration and generation of the ADC map. Getting the patient to co-operate by keeping still during DWI scanning is vital for achieving good slice registration and image quality.

## Quantitative assessment with ADC values

**Fig. 4:**
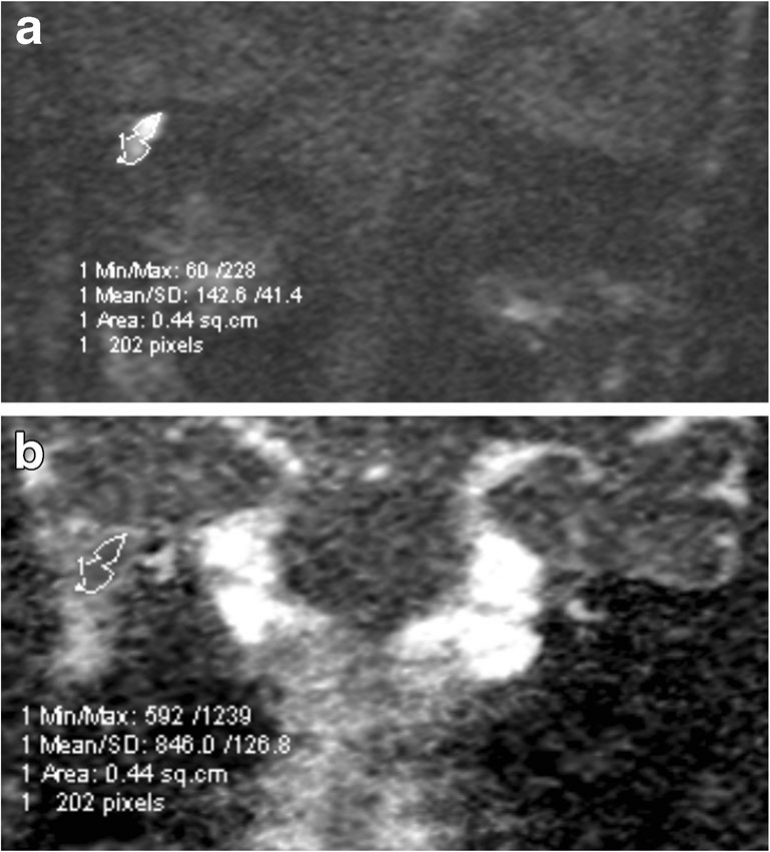
Measuring the ADC value of a lesion. **a** As the lesion was better seen on the b1000 image, a freehand region of interest (ROI) was contoured around the inner border of the lesion. An image was selected so the central part of the lesion was measured to avoid partial volume averaging. The software then maps the ROI on **b** the corresponding ADC map where the ADC value is automatically calculated ADC value 846 x 10^-6^ mm^2^/s. For accuracy, this can be repeated several times and a median reading obtained. In some cases, if the lesion is well seen on the ADC map, the ROI can be placed directly on the map for ADC value calculation

Cholesteatomas have been shown to have a significantly lower ADC value than non-cholesteatomatous soft tissue [12]. The threshold ADC values for differentiating the tissue types need to take into account data acquisition parameters [12, 26]. Once the ADC threshold is established for the scanner and the scanning protocol, it performs well in discriminating cholesteatoma from non-cholesteatomatous soft tissue with comparable sensitivity and marginally higher specificity than the qualitative method and can help navigate beyond some radiological interpretation pitfalls [12]. The ADC value can be measured by simply placing on the ADC map a region of interest (ROI) on the lesion, preferably on an image section that demonstrates the lesion with the highest contrast. For a more accurate measurement, a freehand ROI can be used and contoured around the inner border of the central aspect of the lesion to avoid the edges and partial volume averaging, and a median of several ADC values is obtained [12, 26] (Fig. 4). When residual lesions are small and seen only on two contiguous sections, the mean ADC value can be taken from the two sections for a more representative value [12, 26].

## Diagnostic performance in detecting post-operative cholesteatoma

Following canal wall up mastoidectomy, a mandatory second-look surgery is performed 9–12 months later to detect residual disease. This is because the current literature suggests that canal wall up procedures are associated with rates of residual and recurrent disease of anything up to 36 and 18 %, respectively [3, 27, 28].

There are many studies to date that have evaluated the performance of non-echoplanar DWI in detecting post-operative cholesteatoma [2, 8, 12–23, 25, 29–39]. They are all observational studies with some studies having mixed post-operative and primary cholesteatoma cases in their study samples. The studies include both prospective cohort and retrospective studies, with sample sizes up to 158 patients [38]. The vast majority of the studies demonstrate a sensitivity and specificity between 80 and 100 % in detecting middle ear cholesteatoma. A previous meta-analysis of 10 studies in 2013 reported a sensitivity and specificity of 94 % in detecting middle ear cholesteatoma [5].

The high diagnostic performance of DWI in detecting post-operative cholesteatoma lends support for a non-invasive alternative for reliably detecting post-operative disease. Apart from detecting post-operative cholesteatoma, DWI has also been shown to correlate well with surgery in depicting size and location of the disease [18]. It therefore provides useful information for both operative planning and patient counselling. Despite its high diagnostic performance, the technique is associated with some pitfalls in image interpretation attributed by limitations in the technique, variability in the nature and behaviour of cholesteatoma and characteristics and complexity of the reconstructed post-operative anatomy.

## Pitfalls in sensitivity

**Fig. 5:**
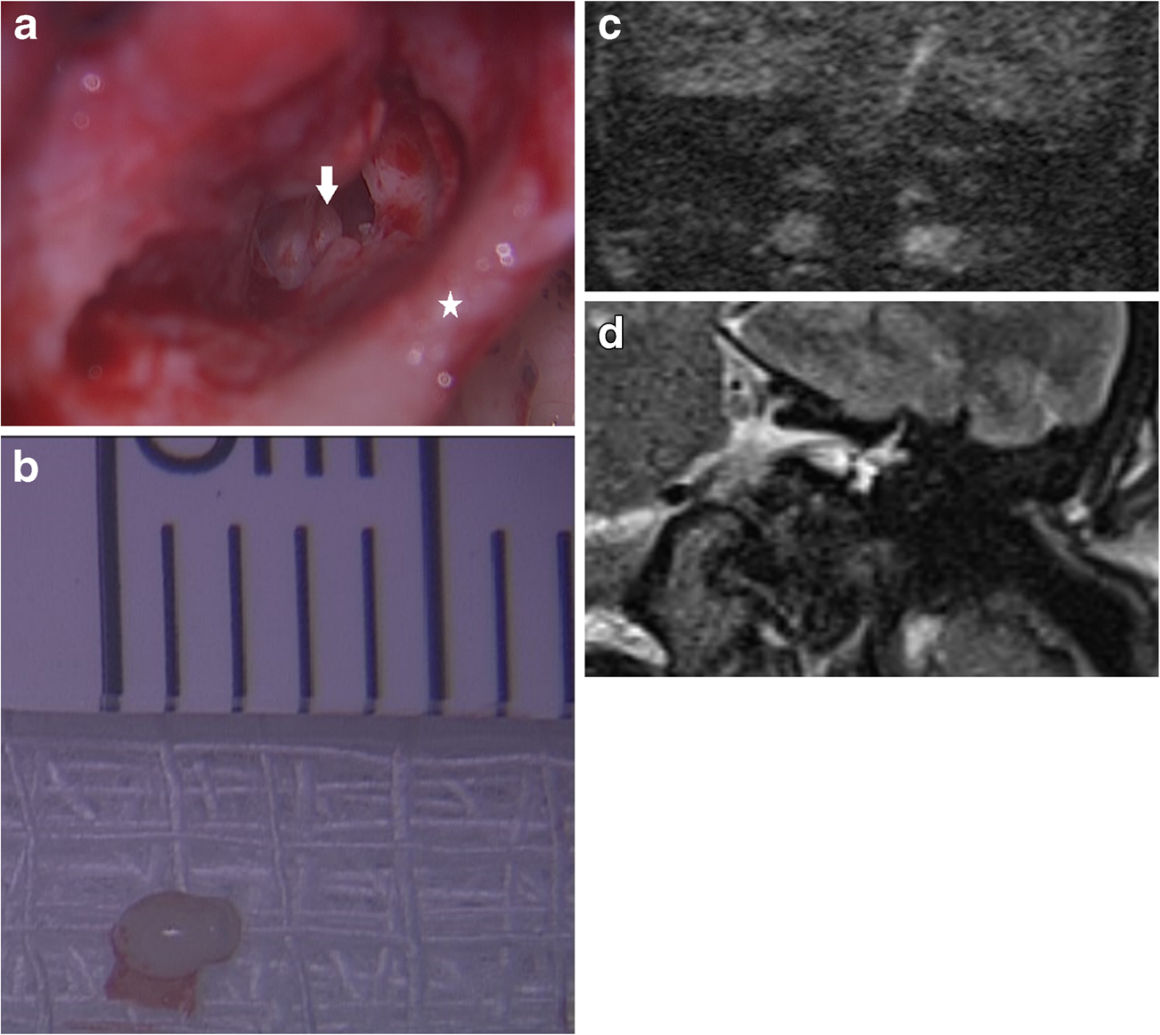
False negative case for cholesteatoma on DWI. **a**, **b** A small 2 mm cholesteatoma pearl (*white arrow*) was found on surgery in the hypotympanum *(star denotes posterior canal wall)*, but was not depicted on **c** the corresponding 2 mm thick coronal b1000 image or **d** T2 weighted image

A high sensitivity is clinically more desirable than a high specificity if DWI were to replace mandatory second-look surgery as it will reduce residual or recurrent cholesteatomas from being missed. The studies have shown consistently reported that DWI is limited in its ability to detect small cholesteatoma pearls less than 3 mm (Fig. 5). Occasionally slightly larger cholesteatoma up to 5 mm can be missed, presumably due to the nature of the cholesteatoma (lack of necessary keratin to return high DWI signal or poor image quality (movement artefact) [3, 18, 38] . There are a few studies in the literature with lower reported sensitivities than expected, this being attributed to a larger proportion in their study sample of cholesteatoma less than 3 mm in size [17, 29]. In addition, no difference was demonstrated between the performance using a 3T scanner [17, 25] and a 1.5T scanner, both equally limited in reliably detecting cholesteatomas less than 2–3 mm. Other less common causes of false negative cases described in the current literature include auto-atticotomy (an epithelial lined sac) [8, 23, 29, 32], aspiration of cholesteatoma on microsuction [23], and patient movement artefacts [37].

## Navigating past the pitfalls in sensitivity

**Fig. 6:**
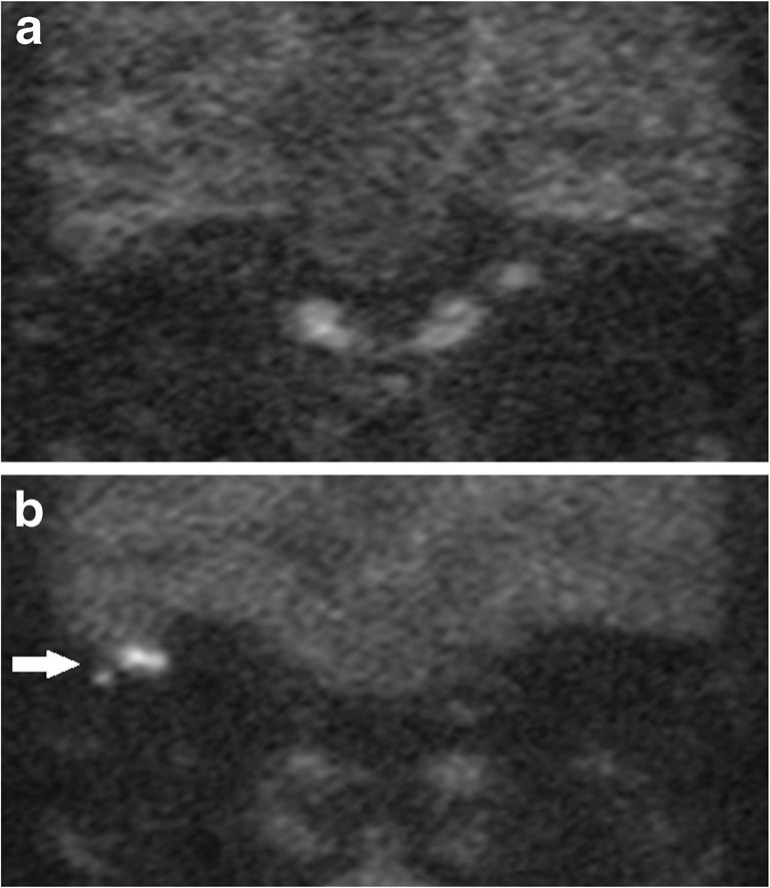
Value of monitoring DWI scans which are negative for the presence of post-operative cholesteatoma. A year following right canal wall up surgery, the DWI scan was negative as shown by the **a** coronal b1000 image. The patient opted for monitoring with DWI instead of second-look surgery as his ear was dry. **b** A follow-up DWI scan a year later showed residual cholesteatoma (*white arrow*), which was confirmed on subsequent surgery

Avoiding a second-look or re-look surgery with a non-invasive technique such as DWI has financial benefits and the advantage of reducing surgical morbidity [3]. Despite the high diagnostic performance of DWI, the inability of a negative DWI scan to entirely exclude the presence of a small cholesteatoma impacts its potential to replace mandatory second-look surgery. When faced with a negative DWI, the decision to perform second-look surgery depends on many factors, including current clinical/otoscopic signs, the extent of initial disease and clinical confidence in surgical clearance at initial surgery, patient preference, and other risk factors of surgery such as patient’s age and co-morbidity [3]. Several authors have proposed a reasonable approach such as replacing second-look surgery in low-risk cases and following them up with serial annual DWI over a reasonable length of time [3, 18, 38]. This will allow time for any small undetected cholesteatoma to grow and accumulate keratin to become sufficiently large to be detectable on DWI (Fig. 6). A recent study demonstrated that in patients with a negative first post-operative follow-up DWI and no clinical signs of post-operative cholesteatoma, a repeat second-follow up scan (performed at least 6 months after first follow-up DWI) was positive or equivocal for disease in 14 out of 45 (31 %) ears, with five ears having cholesteatoma confirmed on surgical exploration [40]. In addition, 2 third-follow-up DWI scans, which were previously negative, turned positive for cholesteatoma and was confirmed on surgery. It is, however, currently unclear what the optimal length and frequency should be for serial follow-up imaging with DWI, and this is still subject of research and discussion. The main drawback to this imaging strategy is the loss of patient to follow-up [3, 18].

Keeping scanning time as short as possible, briefing the patient on the importance of keeping still during scanning and the use of sedation usually helps prevent or reduce movement artefacts and misregistration errors in ADC map calculation [38].

## Pitfalls in specificity

**Fig. 7:**
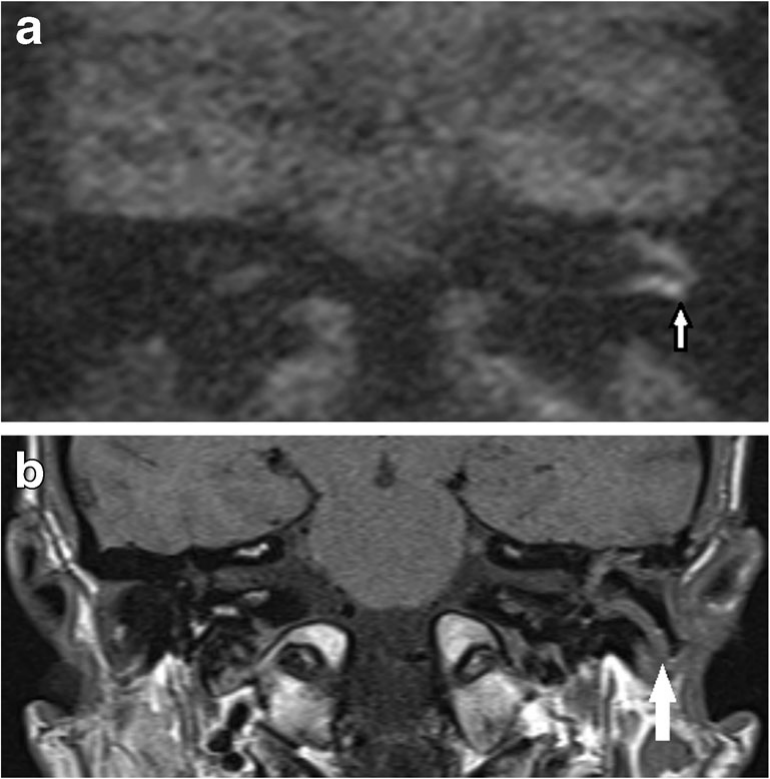
Case of wax in the ear canal (*white arrow*) mimicking residual disease. The **a** b1000 image shows parallel high signal, which corresponded to wax in the adjacent ear canal, localised better on **b** the corresponding non-contrast enhanced T1 weighted image

**Fig. 8:**
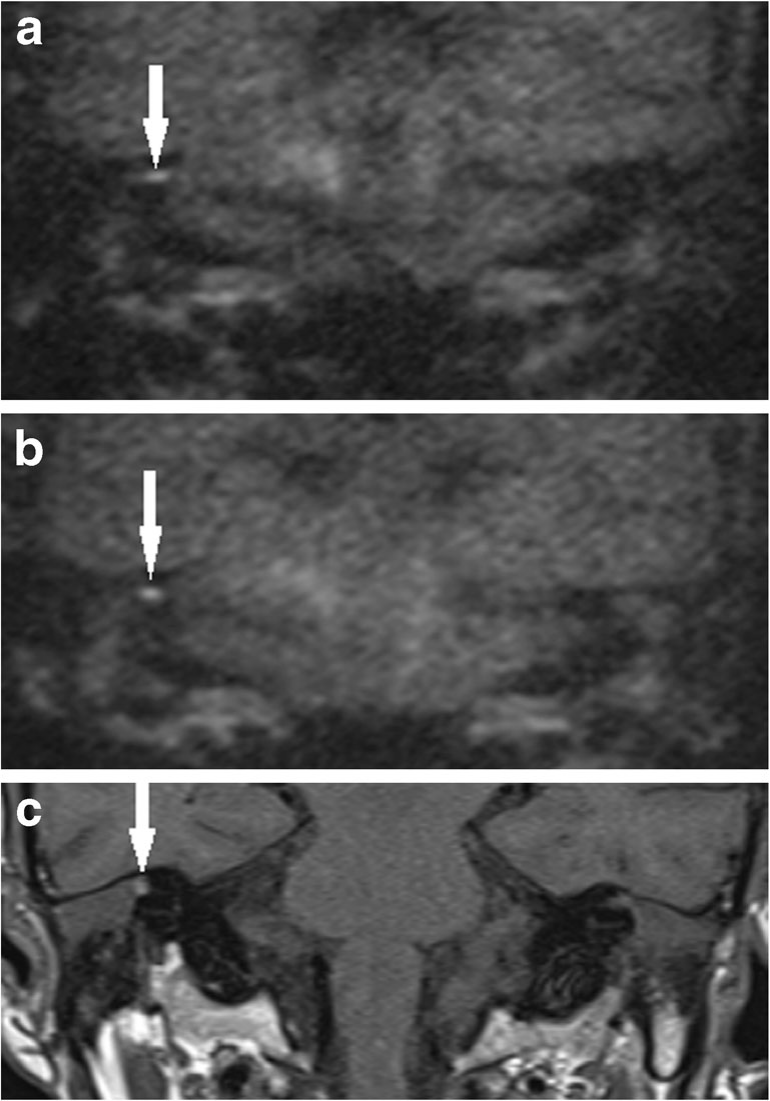
False positive case of proteinaceous fluid in the mastoid remnant. The coronal **a** b1000 image shows a small focus of high signal (*white arrow*) postero-medially at the mastoid remnant, which returned low signal on the ADC map, but was distant from the site of the original middle ear cholesteatoma which was excised with high surgical confidence. At the clinico-radiological meeting, it was decided to monitor this annually with DWI. **b** The subsequent coronal b1000 image showed the high signal focus (*white arrow*) remained unchanged over 2 years making it more unlikely to represent residual disease. **c** The corresponding non-contrast enhanced T1 weighted image showed the lesion (*white arrow*) returned high T1 signal suggestive of proteinaceous fluid given its stability

**Fig. 9:**
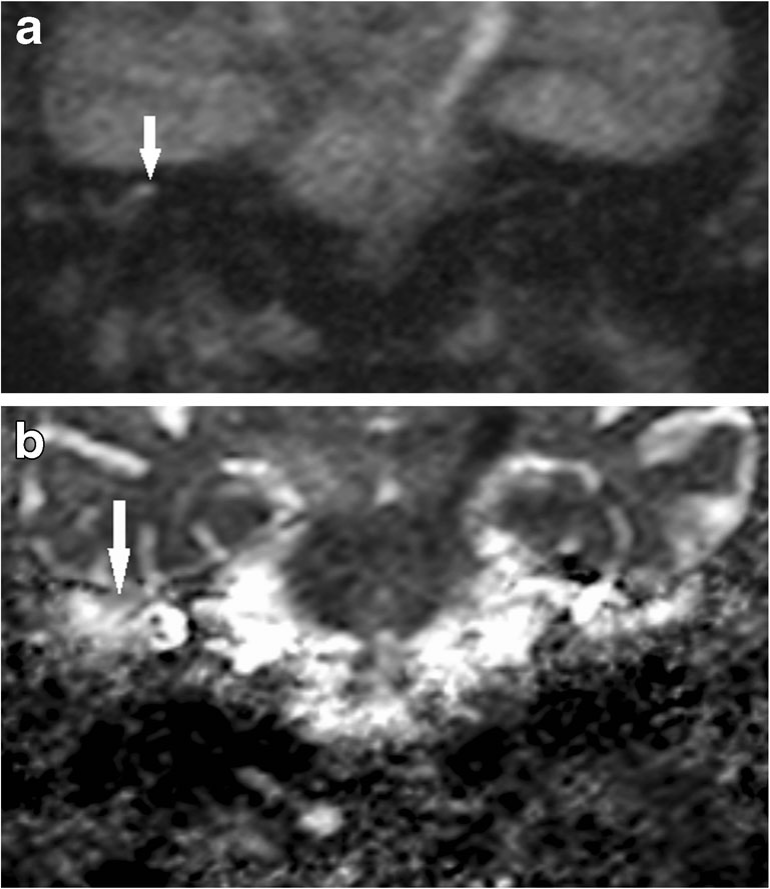
False positive high DWI signal for cholesteatoma. Non-cholesteatomatous tissue (surgically confirmed non-specific inflammation) at the post-operative mastoid remnant, which demonstrated **a** high signal (*white arrow*) on the b1000 image suggestive of residual cholesteatoma. However, **b** the corresponding ADC map demonstrated borderline high ADC signal (*white arrow*) and value *not supporting* the diagnosis of residual cholesteatoma. This case could also have been resolved with a follow-up DWI scan

**Fig. 10:**
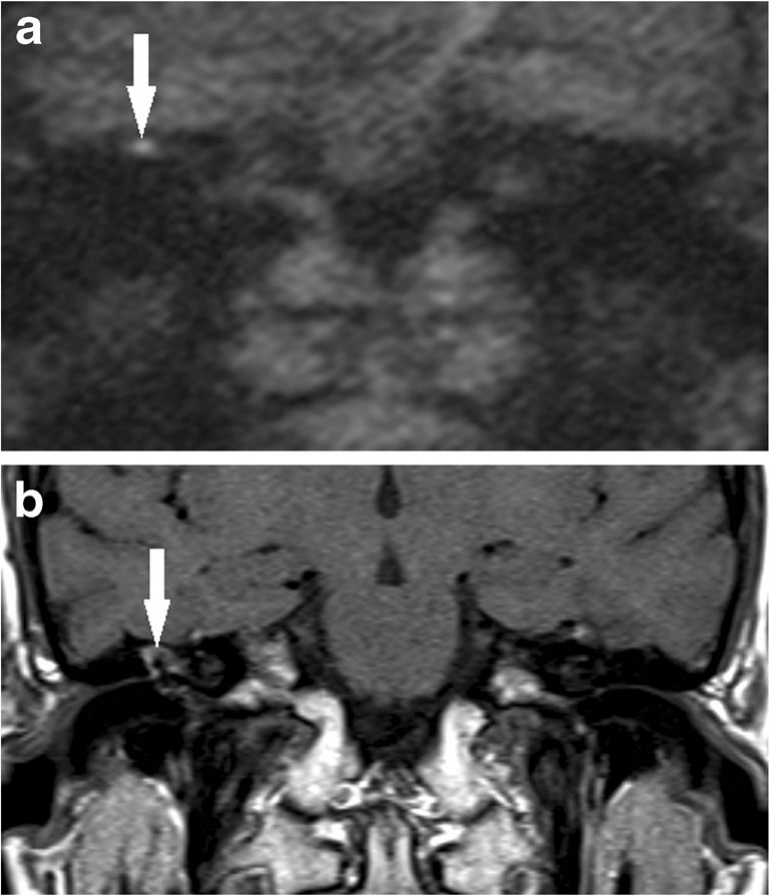
False positive high DWI signal for cholesteatoma. **a** The coronal b1000 image shows high signal at the epitympanum, which raised suspicion for residual cholesteatoma. However, **b** the corresponding non-contrast enhanced coronal T1 weighted image showed high signal (*white arrow*) and the ADC map intermediate signal and value, both *not supporting* the diagnosis of cholesteatoma. Surgery confirmed non-specific inflammation with no cholesteatoma

There is a myriad of false positive cases described in the literature for detecting cholesteatoma using non-echoplanar DWI. Commonly in our experience, wax (Fig. 7) or proteinaceous fluid (Fig. 8) /cysts and non-specific inflammation (Figs. 9 and 10) can return high signal on the b1000 images [3, 12, 22, 23, 32, 35, 38]. They can also be a result of operative materials such as silastic sheet, bone dust/powder [2, 20, 35], and (calcified) cartilage [38]. There are reports of false positive cases from dental braces artefacts [17], tympanosclerosis [38], cholesterol granuloma [22, 38], and squamous cell carcinoma of the external auditory canal [35].

## Navigating past the pitfalls in specificity

To improve diagnostic performance including specificity, image interpretation should be performed in a clear clinical context and not in isolation. A useful way to resolve disconcordant or questionable DWI high signals is for the radiologists to discuss with the otologists, ideally in a joint clinico-radiological meeting. The treatment history, such as type of operation(s) performed and when, the materials used, surgical confidence of clearance, the extent of initial disease (small pearl in the epitympanum or extensive disease into the mastoid) and current ear status (discharging or quiet), and examination are all relevant and important clinical factors to consider. A small focus of dependant high b1000 signal and restricted diffusion posteriorly at the mastoid is unlikely to represent a cholesteatoma pearl if the initial primary disease was a small epitympanic cholesteatoma which was easily and confidently removed on surgery. Indeterminate DWI signals of low radiological and surgical suspicion for cholesteatoma can easily be followed up with DWI over time to ensure stability or resolution and hence exclude the possibility of cholesteatoma with more certainty (Fig. 8). In addition, by providing feedback to the radiologists following surgery, learning is reinforced and hence better and more confident radiological interpretation is nurtured.

**Fig. 11:**
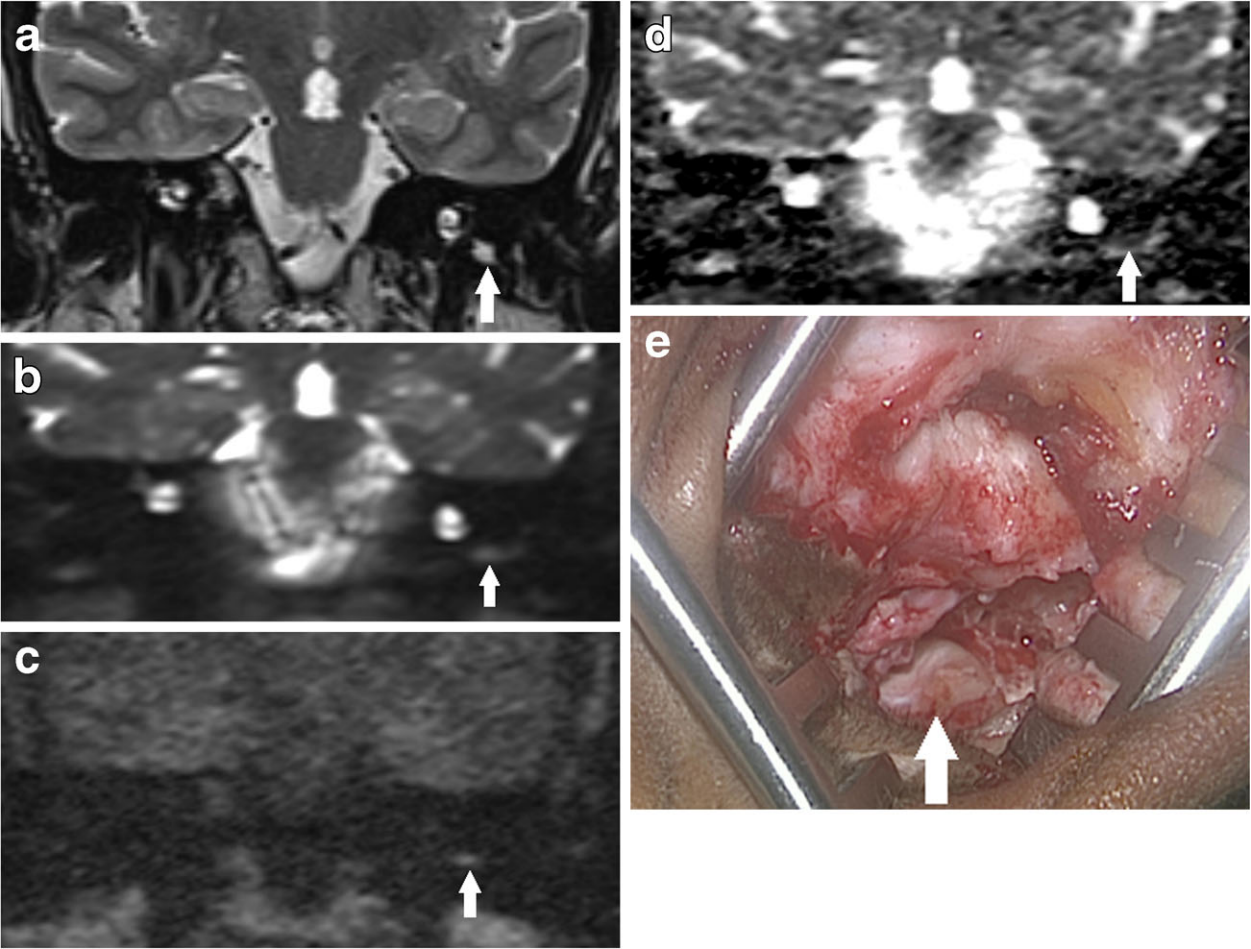
Indeterminate appearance of residual cholesteatoma: Value of ADC. The coronal **a** T2 weighted and **b** b0 DWI images show a small focus of high signal soft tissue at the left hypotympanum, which returns just mildly high signal relative to brain tissue on **c** the b1000 DWI image, raising the possibility of a residual pearl (similar to Fig. 9a). **d** The ADC map demonstrates low signal and value compatible with cholesteatoma, thereby increasing observer confidence. **e** This was later confirmed as a 3–4 mm cholesteatoma at the left hypotympanum on second-look surgery

**Fig. 12:**
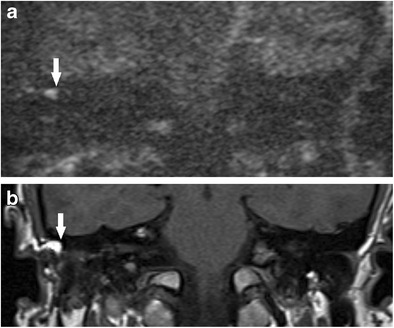
Value of non-contrast enhanced T1 weighted images. False positive high DWI signal (*white arrow*) for cholesteatoma was seen on **a** the b1000 image. **b** The non-contrast enhanced T1 weighted image showed this (*white arrow*) to represent subcutaneous tissue just outside the reconstructed post-operative middle ear cleft. Otoscopic examination of the area is normal and following discussion at the clinicoradiological meeting, a follow-up DWI was arranged. This showed stable appearance

Supplementing qualitative analysis of the DWI images and ADC map with other MRI sequences and ADC values can also help improve specificity [12]. Quantitative analysis using ADC values can provide insight into the nature of the indeterminate lesions because cholesteatoma typically produces significantly lower ADC values than non-cholesteatomatous soft tissue including granulation tissue, proteinaceous fluid or inflammation [12] (Figs. 9 and 10). Not infrequently, the cholesteatoma pearls formed from residual disease are not large enough to return the characteristic higher signal than brain tissue on the b1000 image and instead return intermediate or borderline high signal (Fig. 11). They can pose a diagnostic challenge, but corroborating with its ADC value can help improve the observer’s confidence level in detecting cholesteatoma [12]. T1-weighted images may also help in improving specificity of the DWI images, as some proteinaceous fluid or non-cholesteatomatous soft tissue including cholesterol granuloma can return high T1W signal which is not associated with cholesteatoma [4] (Figs. 8, 10, and 12). The location and configuration of the lesion is also informative. Wax in the ear canal can return linear (often parallel) high signal changes on the b1000 images and low signal and values on the ADC map (Fig. 7). As they can depict anatomy better than the DWI images, the T1W and T2W images are especially useful in improving localisation of the lesion and assessing whether spurious DWI high signals mimicking possible cholesteatoma actually lie within or outside the post-operative middle ear cleft (Figs. 7 and 12). Fusion of DWI images with high resolution CT images can depict clearer localisation of DWI signal of post-operative cholesteatoma in relation to the bony anatomy and aid in surgical planning [41], but this would incur additional radiation exposure.

## Use of DWI following canal wall down surgery

**Fig. 13:**
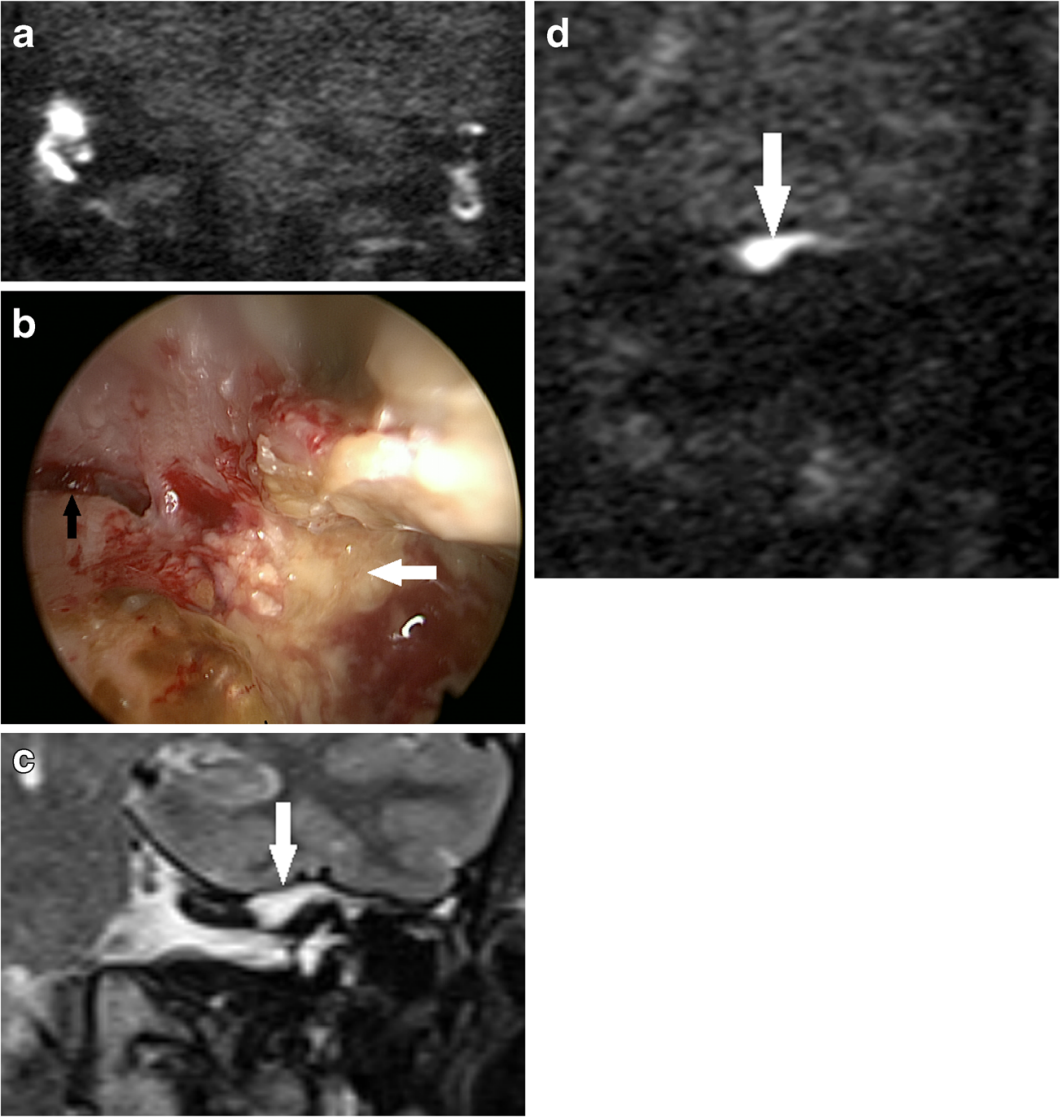
Using DWI following canal wall down surgery. In a patient who presented with persistent otorrhoea after CWD surgery, **a** the DWI b1000 images show high signal in the mastoid remnants bilaterally, which corresponded to infected debris/wax on clinical inspection. **b** On direct clinical inspection of the left mastoid remnant, which showed the infected debris/wax (*white arrow*), a bony defect (*black arrow*) is noted on the medial wall above the bony labyrinth. The defect communicated with extensive residual cholesteatoma hidden deep in the petrous apex, which was demonstrated by high signal soft tissue (*white arrow*) on the coronal **c** T2 weighted and **d** b1000 images

**Fig. 14:**
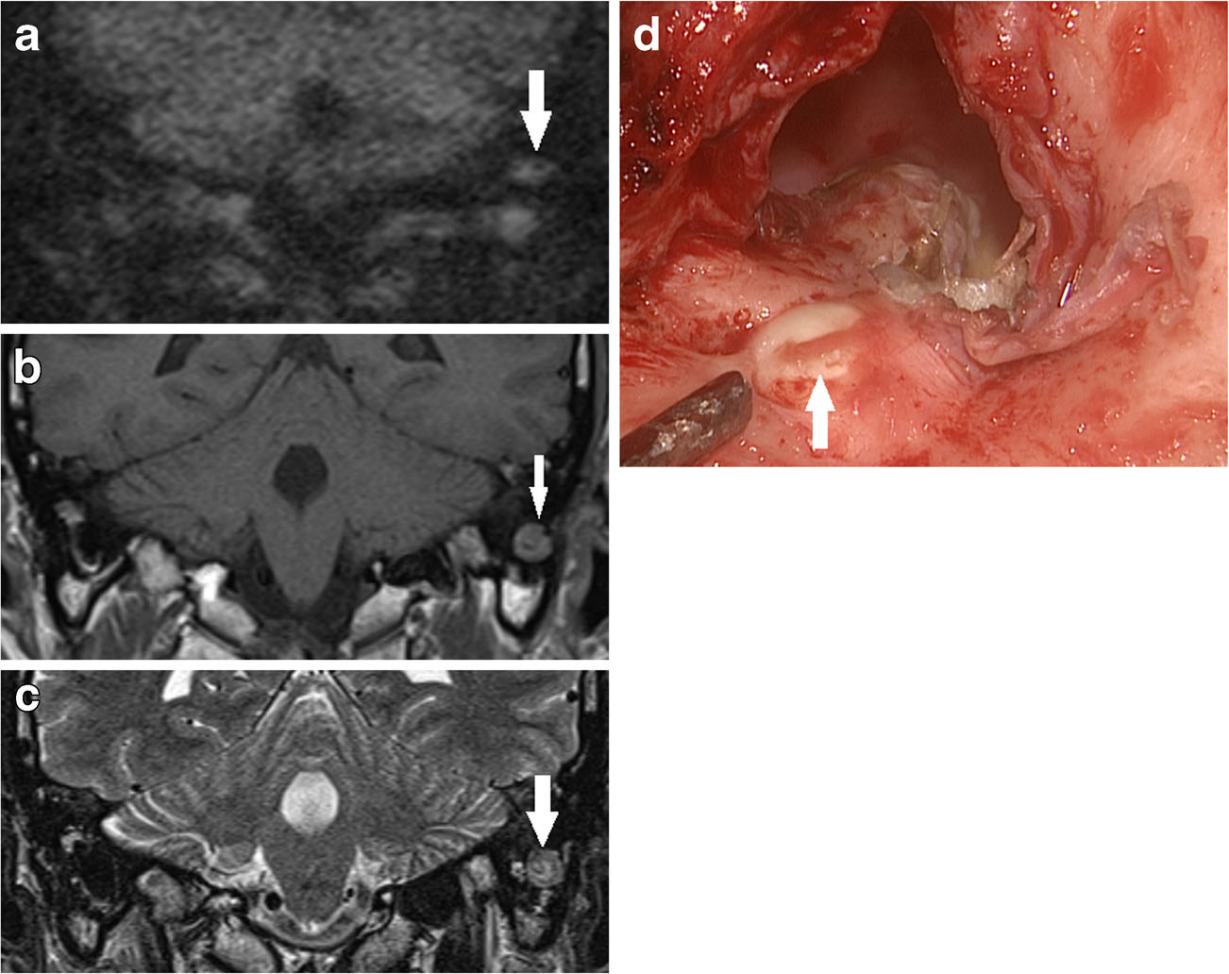
Using DWI following canal wall down surgery. **a** DWI b1000 image depicted residual disease (*white arrow*) at the mastoid tip, also seen as low signal on the **b** corresponding non-contrast enhanced T1 weighted image and a heterogenous, but predominantly high signal on the **c** T2 weighted image. **d** Surgical exploration confirmed residual cholesteatoma pearl (*white arrow*) walled off in the mastoid tip and was not readily visible on direct inspection at the preceding follow-up clinic. The lesion also had low ADC signal and value

The risk of residual or recurrent disease following canal wall down (CWD) mastoidectomy is much lower than that of canal wall up mastoidectomy [3, 5]. When accumulating keratin occurs within a mastoid cavity, it can often be readily detected and successfully cleared with microsuction in an outpatient setting. In addition, wax has a tendency to accumulate in a CWD mastoid cavity, and this can cause false positive signals on DWI (Fig. 13a). However, there are instances where DWI is especially valuable when disease is not readily visible on otomicroscopic examination, such as disease extension into the petrous apex (Fig. 13) or mastoid tip (Fig. 14), disease deep to an obliterated cavity, in a small reconstructed middle ear cavity or medial to an opaque tympanic membrane reconstruction [42]. Close communication between the radiology and otology teams, with clear clinical information, and a clear indication for imaging is crucial.

## Use of DWI in children

CWU surgery is preferred in children because it obviates the potential requirement for lifelong ear care, improves the fitting of hearing aids when required, and is less likely to lead to otorrhoea associated with swimming [3]. Cholesteatoma in children, however, is more aggressive than adult disease and has a higher rate of recurrent and residual disease post-operatively [43] and the otologist is more inclined to perform mandatory second-look surgery to detect disease. Having said this, a recent large prospective observational study comparing 154 adult cases with 54 paediatric cases in detecting post-operative disease, showed similar good diagnostic performance between the two groups [38]. Hence, clinically, DWI can be considered effective in a paediatric setting as in an adult setting, in which many otologists will have more experience. By avoiding radiation exposure and administration of intravenous contrast medium, DWI is especially attractive for imaging children. However, children are less likely to be tolerant of MRI scanning and may require sedation. In our experience, successful scanning can be achieved without the need for sedation, if the clinician explained the essentials of the procedure to the parent and child beforehand.

## Conclusion

Even though non-echoplanar DWI has a high diagnostic performance in detecting post-operative cholesteatoma, it is not without limitations. The pitfalls of image interpretation need to be recognised and managed appropriately by both radiologists and otologists for it to contribute to effective and safer patient care.
